# Relationship between Composition and Toxicity of Motor Vehicle Emission Samples

**DOI:** 10.1289/ehp.6976

**Published:** 2004-07-15

**Authors:** Jacob D. McDonald, Ingvar Eide, JeanClare Seagrave, Barbara Zielinska, Kevin Whitney, Douglas R. Lawson, Joe L. Mauderly

**Affiliations:** ^1^Lovelace Respiratory Research Institute, Albuquerque, New Mexico, USA; ^2^Statoil Research Centre, Trondheim, Norway; ^3^Desert Research Institute, Reno, Nevada, USA; ^4^Southwest Research Institute, San Antonio, Texas, USA; ^5^National Renewable Energy Laboratory, Golden, Colorado, USA

**Keywords:** diesel exhaust, gasoline exhaust, hopane, mutagenicity, PAHs, particulate matter health effects, principal component analysis, semivolatile organic carbon, sterane, toxicity of motor vehicle emissions

## Abstract

In this study we investigated the statistical relationship between particle and semivolatile organic chemical constituents in gasoline and diesel vehicle exhaust samples, and toxicity as measured by inflammation and tissue damage in rat lungs and mutagenicity in bacteria. Exhaust samples were collected from “normal” and “high-emitting” gasoline and diesel light-duty vehicles. We employed a combination of principal component analysis (PCA) and partial least-squares regression (PLS; also known as projection to latent structures) to evaluate the relationships between chemical composition of vehicle exhaust and toxicity. The PLS analysis revealed the chemical constituents covarying most strongly with toxicity and produced models predicting the relative toxicity of the samples with good accuracy. The specific nitro-polycyclic aromatic hydrocarbons important for mutagenicity were the same chemicals that have been implicated by decades of bioassay-directed fractionation. These chemicals were not related to lung toxicity, which was associated with organic carbon and select organic compounds that are present in lubricating oil. The results demonstrate the utility of the PCA/PLS approach for evaluating composition–response relationships in complex mixture exposures and also provide a starting point for confirming causality and determining the mechanisms of the lung effects.

Mobile source emissions are important contributors to ambient air pollution and have been associated with cancer-related and non-cancer-related health effects. Recent work has shown that health effects and ambient air pollution increase with proximity to roadways, suggesting that motor vehicle traffic (engine emissions) contributes a large share to ambient health effects (Nicolai et al. 2003; Pearson et al. 2000; van Vliet et al. 1997; Venn et al. 2001). Two interrelated issues pertaining to the health hazards of motor vehicle emissions continue to present serious challenges to manufacturers, regulatory decision makers, toxicologists, and risk assessors. First, it is important to identify the most important contributors to health risk among the myriad physical–chemical species contained in emissions. Second, it is important to be able to estimate changes in health risks that will result from changes in the composition of emissions. Both issues are important for ensuring that the most health-relevant components are controlled and that technologic strategies for meeting emissions regulations reduce rather than increase hazards. The current knowledge base does little to support such judgments because there have been few direct comparisons of the health effects of different types of emissions. Moreover, except for bioassay-directed fractionation schemes that have identified nitro-polycyclic aromatic hydrocarbons as major drivers of bacterial mutagenicity, few approaches have been used to determine the chemical species driving the health hazards of complex emissions.

We have reported the results of studies in which both bacterial mutagenicity (by Ames tests) and lung toxicity assays of inflammation, cytotoxicity, and lung tissue damage (Seagrave et al. 2002) were assessed to rank the toxic potency of motor vehicle exhaust samples of different chemical composition. That work used combined suspensions of particulate material (PM) and vapor-phase semivolatile organic carbon (SVOC) samples collected from a range of in-use (rented from owners) gasoline- and diesel-powered vehicles, including “high-emitting” vehicles. Preliminary studies showed that it was important to include the vapor-phase SVOC because it comprised a large portion of the mass [defined by gravimetric weight as described by Seagrave et al. (2002)] emitted from some vehicles and could contribute substantially to toxicity as evidenced by evaluation of lung inflammatory responses of separated PM and SVOC samples collected from a traffic tunnel (Seagrave et al. 2001). The samples were combined into seven distinct groups of gasoline and diesel-powered vehicles. There was a 5-fold range in the potency of the samples for lung injury, and samples from high-emitting vehicles (both diesel and gasoline powered) had the highest pulmonary toxicity per unit of mass. There was also up to a 10-fold difference in the bacterial mutagenicity among these samples, with no clear difference between the potency of diesel exhaust and gasoline exhaust on the basis of mutations per milligram of sample.

We also reported the results of detailed compositional measurements of the exhaust samples described above (Zielinska et al. 2004). In the present work, we applied multivariate data analysis to determine the relationship between composition and health response. We selected a statistical approach that had been successfully used to determine the key components of organic extracts of diesel exhaust particles causing mutations in bacteria (Eide et al. 2001, 2002; Sjogren et al. 1996) and aryl hydrocarbon receptor induction (Sjogren et al. 1996). The combined principal component analysis (PCA) and partial least-squares regression (PLS; also known as projections to latent structures) approach allows analysis of the similarities and differences in the specific health responses (e.g., mutagenicity vs. lung toxicity) relative to composition. The product of this work is an assessment of the ability of the PLS to “explain” the composition–response relationship and an indication of which chemical compounds in the exhaust samples were most strongly associated with the health response.

## Materials and Methods

In this article we summarize only the general approaches used for collection, chemical characterization, and toxicity evaluation of vehicle exhaust samples that have been reported in detail elsewhere (Seagrave et al. 2002; Zielinska et al. 2004). The classifications of vehicle samples, chemical/physical classes measured in these samples, and the toxicologic evaluations conducted (including health response category) are summarized in Table 1.

### Emission samples.

Particle and vapor-phase SVOC fractions were collected using filters (for particles) and polyurethane foam/XAD-4 resin traps (for vapor-phase SVOC), respectively, from diluted, fresh emissions from vehicles operated on chassis dynamometers over the unified driving cycle at the Southwest Research Institute (San Antonio, TX, USA) (Whitney 2000, Zielinska et al. 2004). The unified driving cycle is a high-speed, rapid-acceleration test cycle, consisting of a 300-sec cold start phase followed by a 1,135-sec hot stabilized phase, and a 300-sec hot start phase, which is a repeat of the first phase. The maximum speed employed in the cycle was 67.2 mph, with a maximum acceleration of 6.9 mph/sec. Five vehicles or composite groups of vehicles were included: a group of five “normal-emitting” gasoline vehicles (G); a group of three normal-emitting diesel vehicles (D); two “high-emitting” single gasoline vehicles emitting white (WG) or black (BG) smoke; and a single high-emitting diesel vehicle (HD). Specific emission rates for these vehicles are reported elsewhere (Zielinska et al. 2004). All vehicles were in-use light- or medium-duty passenger cars, pickup trucks, or vans, ranging from 1976 to 2000 model years and were tested with fuel and crankcase oil as received (recruited in San Antonio, TX, USA). The normal-emitting groups were sampled while operating both at room temperature and at approximately 30°F (~ −1°C; G_30_, D_30_).

### Chemical characterization of emission samples.

The chemical composition of the particle and SVOC fractions of each of the seven samples was analyzed at the Desert Research Institute (Reno, NV, USA) as described elsewhere (Zielinska et al. 2004). Analyses included temperature fractions of organic and elemental carbon, elements (metals and associated analytes), inorganic ions (sulfate, nitrate), and speciation of resolvable organic compounds. The temperature fractions were obtained using the IMPROVE thermal carbon analysis technique (Chow et al. 2001), in which eight discrete fractions of carbon are vaporized in a changing temperature and helium/oxygen atmosphere. The temperature fractions are grouped into four “organic” and four “elemental” carbon designations as shown in Table 2. Although these are not explicitly chemical fractions (related to both chemical and physical properties), they provide data on differences among the emission samples and have been used in source apportionment modeling studies to differentiate among motor vehicle and other types of emissions (e.g., Maykut et al. 2003; Watson et al. 2002). The organic species measured focused on components that have been used in previous studies to illustrate differences among motor vehicle and other types of emissions. The classes of organic compounds included polycyclic aromatic hydrocarbons (PAHs), ranging in molecular weight from 128 to 300 Da, a mass range that spans from compounds that are considered to be exclusively gases to species found exclusively in the particle phase. Several subclasses of PAHs, including oxygenated PAHs (oxy-PAHs: ketones, aldehydes, quinones), nitro-PAHs, and sulfur-containing PAHs were measured. Hopanes and steranes, two classes of compounds that are found in lubricating oil (McDonald et al. 2003; Rogge et al. 1993), were also measured. A total of 184 composition variables were measured.

### Toxicologic evaluations.

Aliquots of the PM and SVOC extracts (in acetone) were provided (by Desert Research Institute) to the Lovelace Respiratory Research Institute for toxicity testing [description of sample extraction and handling described briefly below and in more detail by Seagrave et al. (2002)]. The PM and SVOC samples were combined (original PM and SVOC were extracted separately) and either mixed with *Salmonella* culture media (Ames bacterial reverse mutation assay) or instilled into lungs of F344/CRL rats (Charles River Laboratory, Wilmington, MA, USA) over a range of total mass (PM plus SVOC) doses (Seagrave et al. 2002). Responses in rat lungs were evaluated at 4 hr (the cytokine MIP-2) or 24 hr (all other responses) after dosing, as described previously (Seagrave et al. 2002). Lungs were removed and weighed, and then cells, protein, enzymes, and chemical mediators of inflammation were measured in bronchoalveolar lavage collected from the right cardiac, diaphragmatic, and intermediate lobes. The left lung was then fixed and examined by light microscopy for histologic evidence of inflammation and tissue damage. In all, 11 lung response variables were measured. Bacterial mutagenicity was evaluated in Ames tester strains TA98 and TA100, both with and without metabolic activation by a liver micro-some preparation (S9) (Seagrave et al. 2002). Dose–response relationships were analyzed for each variable and for each emission sample, and (toxic) potency factors were derived from these analyses (Seagrave et al. 2002).

### Normalization of data for multivariate data analysis.

Data were normalized to weight fraction before statistical analysis by dividing the composition values by the sum of PM and SVOC mass (i.e., composition per unit of total mass). PM was the mass determined on the filter and extracted into solution. As discussed previously (Seagrave et al. 2002), the extraction protocol used to transfer PM into suspension involved agitation, gentle brushing, and sonication in acetone. Analysis of aliquots of the particle extracts was used to measure the recovery of the mass of material in solution compared with the mass weighed on filters before extraction. The recovery for PM was 80–100% for gasoline exhaust samples and 65–70% for diesel exhaust samples. The decreased extraction efficiency for the diesel exhaust samples was likely caused by difficulty in removing elemental carbon from the filters. The SVOC mass was determined by gravimetric analysis of spikes of extracts that were evaporated to dryness to remove the solvent (acetone). Because the compositional data are not reported as weight fractions elsewhere (Zielinska et al. 2004 reported emission rates), we include discussion on the mass composition of the samples and also include the data as Appendix 1 of this report.

### Multivariate data analysis (pattern recognition and prediction).

The compositional data were structured in an X-matrix with one row per exhaust sample (i.e., a total of seven rows) and one column per predictor variable (initially, 184 compositional parameters). The mutagenicity and lung toxicity data were structured in a Y-matrix with seven rows and one column per response variable (i.e., a total of 15 responses). Multivariate data analysis was performed with Simca-P 10.0 (Umetrics, Umeå, Sweden). PCA (Jackson 1991) was performed on the X-matrix to evaluate similarities between mixtures and on the Y-matrix to group responses. PLS was used for the regression modeling to correlate the measured responses to the compositional parameters (Wold et al. 1984). PLS was used for the regression modeling because it overcomes the problems of intercorrelated predictor variables and data matrices where the number of variables exceeds the number of samples (Kettaneh-Wold 1992; Kvalheim 1989).

The purpose of PCA is to define “structure,” or patterns, in data that exist in multiple dimensions. Both the PCA and PLS techniques use the same basic data simplification principles by projecting linear planes (or hyperplanes) into a multidimensional grouping of data (Kettaneh-Wold 1992; Kvalheim 1989). A principal component or a PLS component is a straight least-squares regression line (or plane) through the sample points in the multidimensional space (Sjogren et al. 1996). Each component will “explain” a portion of the variance in the data set. Typically, multiple components are required to explain most of the variance. However, it is desirable to have few principal or PLS components relative to the number of samples for optimal confidence in the outcome of the analysis.

The primary difference between PCA and PLS is that PCA is performed on one data matrix (e.g., X or Y) and PLS evaluates both (X and Y) simultaneously to both develop a predictive model (e.g., predict Y from X) and to evaluate relationships between specific X and Y variables (e.g., which chemicals covary with toxicity?). PCA is first used to identify characteristics of data in either the X- or Y-matrix. The principal outcome of this analysis is the identification of data that “cluster” together similarly and thus are assumed to have a systematic relationship. This application of PCA is illustrated in “Results” by the finding that mutagenicity and pulmonary toxicity variables did not cluster together but that variables within each category did cluster together. This indicated that separate PLS models would be needed for mutagenicity and toxicity.

A PCA analysis was first conducted on the 7 × 184 X-matrix to evaluate similarities between samples by “score plots” and on the 7 × 15 Y-matrix to determine groupings (similarities) among response variables by “loading plots.” This grouping was used to segregate the response variables into covarying groups of responses that could be analyzed by PLS. PLS was initially carried out with all 184 predictor variables; however, because of the low number of samples, PLS had to be carried out on subsets and groups of predictor variables, as explained in “Results.”

Before analyses, the data were mean centered and scaled to unit variance as described previously (Jackson 1991; Wold et al. 1984). Data distributions were evaluated and determined to require no further normalization (e.g., log transformations) before analysis. The results of the PLS analysis were evaluated in terms of both goodness of fit (*R*^2^, analogous to Pearson correlation coefficient) and goodness of prediction (*Q*^2^, determined by cross-validation procedures described in Appendix 2). Each response end point was modeled individually (15 total), and the model results were evaluated by cross-validation procedures. To ascertain that the overall PLS models contained systematic (nonrandom) associations, we validated the models by performing PLS after randomizing (reordering) the values in the Y-matrix as described previously (Eide et al. 2001). This validation procedure is referred to as validation by response permutation (van der Voet 1994). A more detailed description of the validation approach and an example for the validation of one model are included in Appendix 2.

## Results

### Composition of emission samples.

**Figure 1:**
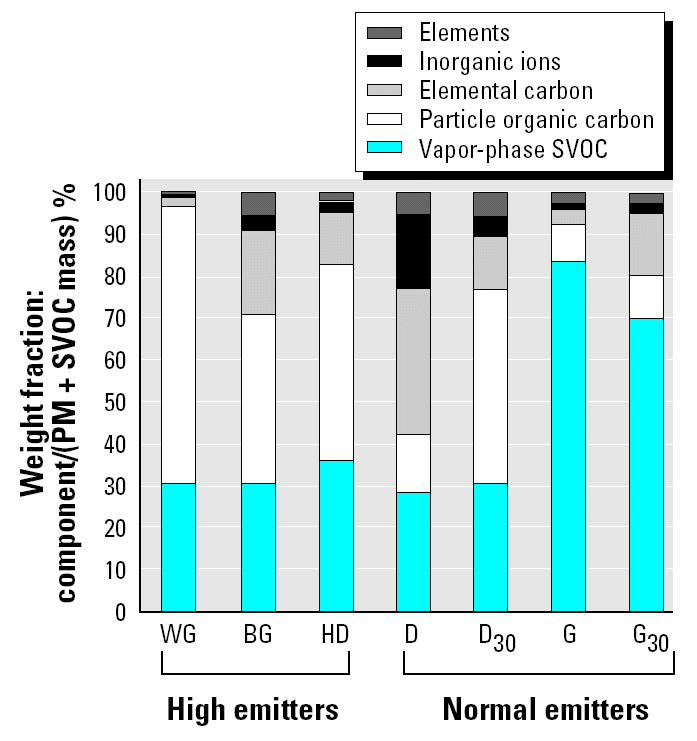
Composition of engine exhaust samples normalized to weight fraction. Individual components were divided by the concentration of total particle and SVOC mass. The sum of particle and SVOC mass equals 100%.

The mass composition of the emission samples is summarized in Figure 1. These results have been reported elsewhere (Zielinska et al. 2004) but only in units of mass/mile traveled. The normal-emitter and black-smoker gasoline samples were composed primarily of vapor-phase SVOC mass, whereas the others were composed primarily of PM. The PM composition ranged from approximately 20 to 95% organic carbon, with no obvious distinction in the proportion of organic carbon between diesel- and gasoline-powered vehicles. This plot does not portray the differences in specific organic classes; the data for individual chemical species are reported in Appendix 1.

The proportions of elements and PAH compounds among these samples were variable, and there was no clear difference in the classifications of vehicles (e.g., high emitter vs. normal emitter or gasoline vs. diesel) that emit higher proportions (as a weight fraction) of any of these classes. In contrast, the higher-emitting vehicles clearly showed higher proportions of the hopane and sterane compounds (components of lubricating oils).

### Principal component analysis.

**Figure 2:**
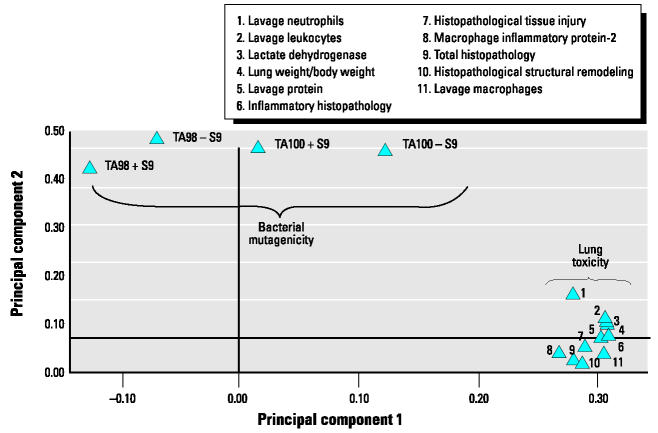
Loading plot showing the groupings among the 15 toxicity measurements. Measurements grouped together responded similarly to the exhaust samples, and their proximity reflects the degree of similarity of responses. Separation of mutagenicity and lung toxicity groups suggested that they responded to different chemical components.

The loading plot shown in Figure 2 was obtained from PCA on the toxicity data and shows the clustering of the 15 different toxicity measurements according to their similarity in responses to the exhaust samples. The 11 lung toxicity responses clustered together in one group, whereas the four bacterial mutagenicity responses occurred at different spots. This indicated that the lung toxicity responses were associated with similar chemical components and that these components were different from those associated with the mutagenicity responses. As a consequence, regression modeling with PLS was done with the 11 lung toxicity end points simultaneously (it is advantageous to use multiple responses because they will support one another in the model), and the four mutagenicity end points were modeled separately.

### PLS analysis of lung toxicity data.

The goal in developing the PLS model was to explain the most variation in the data using the smallest number of PLS components. Initially, PLS was performed with all 184 compositional variables versus the 11 lung toxicity responses. Although it was possible to obtain a PLS model with high *R*^2^ and relatively high *Q*^2^ with all 184 predictor variables, validation by response permutation showed that the overall PLS model could be due to chance because of the large number of compositional variables relative to the relatively small number of samples. To alleviate this, we performed the PLS analysis after grouping most of the individual compositional variables by chemical class (e.g., hopanes) or subclass (e.g., two-ring PAHs). The number of variables was reduced from 184 to 34, and PLS was carried out with these 34 variables versus the 11 lung toxicity responses. The resulting PLS model performance was acceptable (*R*^2^ = 0.95; *Q*^2^ = 0.35) with only two PLS components and was hence used only to obtain first-pass indication of which groups of compounds associated (covaried) most strongly with the lung toxicity responses. According to loadings and PLS regression coefficients (not shown), the variables that associated most strongly with the 11 toxicity responses were particulate organic carbon, select thermal fractions of the carbon analysis, and the hopane and sterane classes of compounds.

**Figure 3:**
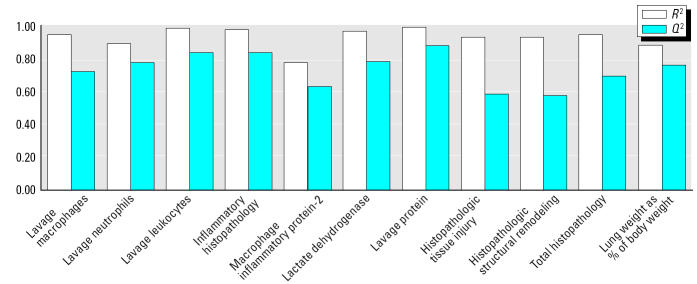
Goodness of fit (*R*^2^) and prediction (*Q*^2^) from the PLS model (68 variables and 11 responses).

**Figure 4:**
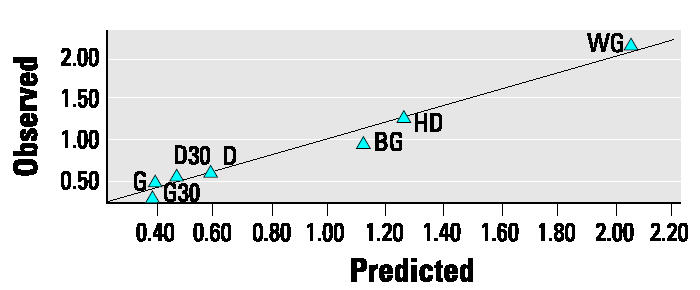
Observed versus predicted histologic inflammation, showing good predictive performance of the PLS model. *R*^2^= 0.97.

The 34-variable PLS model was followed by a PLS model in which some compositional variables were ungrouped into their individual compounds (the hopanes and steranes). This gave a final 68-variable X-matrix (Table 2) that performed well (Figure 3 shows performance for each lung toxicity measurement; overall model performance: *R*^2^ = 0.93; *Q*^2^ = 0.72), accounting for approximately 70% of the variation in the data by just two PLS components (53 and 15% by the first and second PLS components, respectively). Each of the 11 toxicity response PLS models showed satisfactory-to-excellent performance in the validation by permutation tests (results of validations not shown, except the example given in Appendix 2). The model performance indicators for each lung response category (Figure 3) indicated that the model had better predictive capability for direct measures of inflammation (e.g., cell count, histopathology) than for indirect indicators (e.g., MIP-2). An example of the high quality of the model prediction is shown in Figure 4, which illustrates the observed versus predicted response for histologic evidence of lung inflammation.

**Figure 5:**
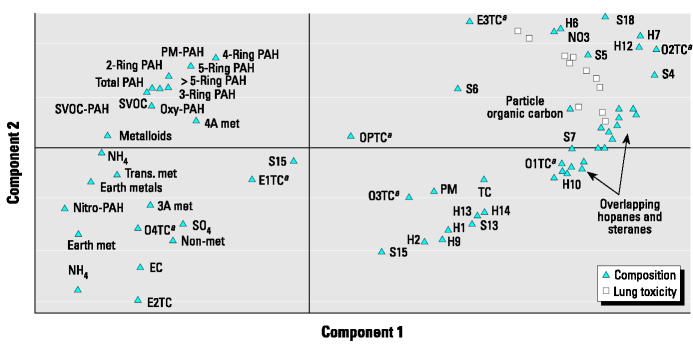
Loading plot showing the lung toxicity and 68 chemical components for the first and second PLS components. Proximity to the lung toxicity responses reflects the strength of association (degree of covariance) of individual chemical components to the response. See Table 2 for abbreviations. ***^a^***Carbon analysis fractions.

Once the predictive model was determined, the strength of association (PLS loadings) between the chemical components and the individual lung toxicity responses was evaluated in a loading plot (Figure 5). This plot, analogous to the plot shown in Figure 2 for the 15 toxicity variables, shows the clustering of toxicity and chemical component variables, illustrating the chemical components that were most closely associated (covaried) with lung toxicity. The plot combines the covariance from the two PLS components that were required for the 68-variable model. The chemical variables have been abbreviated or grouped in the plot, and the full names associated with the abbreviations are given in Table 2 (the abbreviations give an indication of the chemical class). The components that had the strongest association with lung toxicity were most of the hopanes, steranes, and particle-phase organic carbon. The hopanes and steranes are compounds that are found in crude oil and are thus emitted as part of the crankcase oil emissions. These compounds are derived from the diagenesis of plant materials (e.g., conversion of phytosterols to steranes). Their characteristic structures have been described elsewhere (e.g., Rogge et al. 1993). The analysis of fuel and crankcase oil collected from the vehicles studied here (reported in Zielinska et al. 2004) showed that the hopanes and steranes were in high concentrations in oil (as expected) and only trace amounts of the steranes were observed in fuel. High-oil-burning vehicles will also show large amounts of particle-phase organic carbon. The most volatile thermal fractions from the carbon analysis along with one elemental carbon temperature fraction and nitrate also covaried with the lung toxicity responses. Other components, namely, the metals and PAHs, had little or no correlation with the lung responses.

### PLS analysis of mutagenicity data.

**Figure 6:**
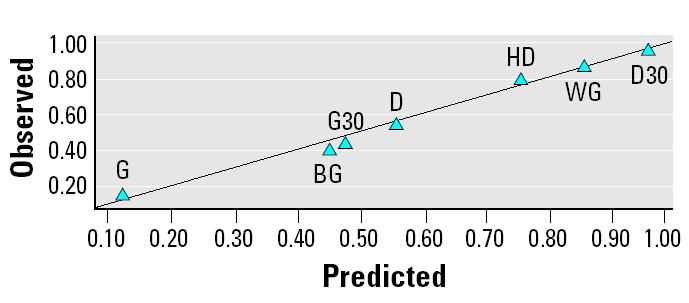
Observed versus predicted mutagenicity of TA100 (without S9), showing good predictive performance of the PLS model. *R*^2^= 0.98.

PLS of the mutagenicity data using either the complete set (184) or the first reduced set (34) of chemical variables in the X data matrix was performed without satisfactory results. The 34-variable data set grouped together the chemical components that were known, based on previous studies, to be mutagenic. However, grouping by compound classes did not reveal associations between composition and mutagenicity and did not yield satisfactory performance in the PLS model. A separate strategy for configuring the X data matrix was adapted that ungrouped the individual nitro- and oxy-PAHs known to be direct mutagens and used them in a reduced data set of 23 variables (Table 3). The best model performance (*R*^2^ = 0.98; *Q*^2^ = 0.73) was obtained with these variables applied to the TA98 and TA100 strains without S9 metabolic activation. Figure 6 shows the observed versus predicted mutagenicity with this PLS model for strain TA100. The models for TA98 and TA100 could explain approximately 60% of the variation with three PLS components. In contrast, PLS models did not perform well for TA98 and TA100 strains with metabolic activation (not shown). This was not surprising because most of the mutagens that have been implicated in engine exhaust are direct acting (e.g., do not require metabolic activation). In addition, the presence of S9 may suppress mutagenicity by inactivating or adsorbing certain mutagens (Shah et al. 1990).

**Figure 7:**
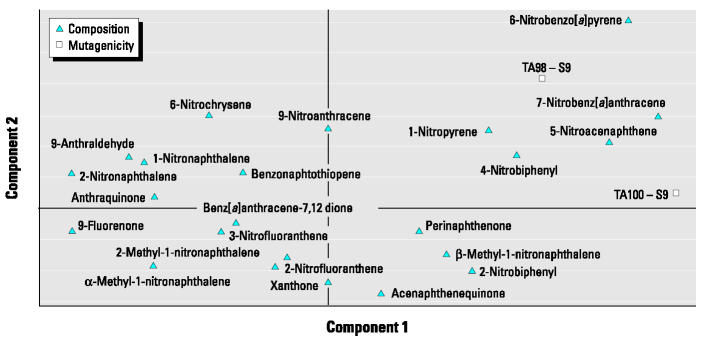
Loading plot showing the mutagenicity and chemical components for the first and second PLS components. Proximity to the mutagenicity responses reflects the strength of association (degree of covariance) of individual chemical components to the responses.

Figure 7 shows the loading plot with combined mutagenicity and chemical variables. Similar to what was expected based on the known mutagenicity of these compounds, the particle-bound higher-molecular-weight nitro-PAH compounds had the highest association with mutagenicity, whereas most of the oxy-PAHs and volatile nitro-PAHs had poor or no association. The similarity between the PLS model associations identified in this study and chemical components that were previously known to drive mutagenicity helped validate the PCA/PLS approach for evaluating composition–response relationships for lung toxicity, for which composition–response relationships were not known in advance.

## Discussion

The present study represents a step toward a better understanding of the physical–chemical components of engine emissions presenting the greatest lung health hazards. There is growing recognition of the need to develop a more integrated understanding of the air quality–health relationship (Mauderly 2003; National Research Council 2001), but disentangling the relative roles of air contaminants in complex environmental pollution and source emissions has progressed slowly. Except for the biodirected fractionation approach that identified certain nitro-PAHs as driving bacterial mutagenic responses, there has been little progress in determining the specific species causing the effects of physically and chemically complex combustion emission mixtures. Most epidemiology and toxicology has focused on specific pollutants (e.g., unspeciated PM or nitrogen dioxide) or treated complex emission exposure atmospheres as a single material. Studies comparing the effects of filtered and unfiltered emissions (Maejima et al. 2001) or the effects of the elemental carbon and extractable organic fractions of diesel soot (Nel et al. 2001) are examples of simplified biodirected fractionation but fall far short of testing the roles of the full range of emission species. Epidemiologists commonly employ multivariate analyses involving multiple environmental pollutants, but have data for only a few pollutant species and usually focus on determining the influence of copollutants on estimates of the effects of the single pollutant (or class, e.g., PM) of chief interest (Samet et al. 2000). In a study conceptually more similar to the present study, Wellenius et al. (2003) applied multivariate regression modeling to data from multiple exposures of dogs to concentrated ambient air PM to identify an association between silicon and cardiac effects, but studied only the PM fraction of pollution and did not have data on speciated organic compounds. There are no previous reports of the use of multivariate analyses to disentangle the roles of both the vapor and PM organic phases of engine emissions.

This study, although certainly an over-simplification of environmental exposures to inhaled emissions, demonstrates that PCA/PLS has potential for exploring complex exposure composition–health response associations, given a suitable data set. The utility of this approach in identifying putative causal agents in diesel exhaust samples had been demonstrated but with only a single health response (mutagenicity) and a larger number of samples (Eide et al. 2002). A challenge in applying PLS in the present study was the inclusion of many health responses (15) and composition variables (184) but only seven samples—a very practical situation in view of the limited sample (or exposure) number and diversity typical of environmental studies. The approach worked well largely because of success in grouping covarying composition and response variables and reducing their relationships into a number of principal components smaller than the number of samples. Grouping compositional components by class, however, has the disadvantage of making the often-false assumption that all species within the class are equally toxic per unit of mass or that the proportions among the grouped compounds are similar. This assumption was certainly not true for mutagenicity, in which case total nitro-PAH mass was poorly predictive, but foreknowledge of the mutagenicity of particular species allowed development of a more focused and highly predictive model. In the absence of little previous information on the contributions of individual components, as was the case for lung toxicity, iterative approaches to grouping the composition into classes and disaggregating classes into individual compounds can be used to explore and optimize models. The small number of samples also raised the possibility that apparently meaningful composition–response relationships could reflect random (nonsystematic) statistical associations. The cross-validation and confirmatory steps were critical to developing confidence that the associations portrayed by the models having the best fit and predictive performance were in fact systematic (nonrandom). Overall, the results suggested that PCA/PLS can be useful for identifying composition–response associations for complex exposures even when the number of exposure cases is small. An alternative to grouping and variable selection is hierarchical PLS [described by Wold et al. (1996)], which was used (not shown) to confirm the conclusions of the PCA/PLS results presented in this article.

The use of collected and processed samples, wherein acetone was used to extract species from the collected exhaust material, was a limitation of this study. First, the exhaust collections account for only a portion of the exhaust. Although attempts were made to quantitatively remove 100% of the PM from the filters, only approximately 65–70% of the PM from diesel exhaust samples with high amounts of inorganic carbon could be removed. Although the vapor-phase SVOCs were collected, the samples did not include the most volatile vapor and gas components of the exhaust. In addition, it is known that chemical artifacts can be induced during sample collection and processing (Arey et al. 1988), and it is possible that there were potentially important compositional differences between the collected samples used for this study and the original emissions. However, confidence in the present results derives from the fact that the hopanes and steranes having the strongest associations with toxicity are not formed by artifact, are chemically stable (not prone to decomposition), and are known components of lubricating oil. Thus, although the roles of components that might have been lost during sample processing could not be tested, the components most strongly associated with the lung responses were extremely unlikely to be artifacts.

Instillation of extracted material into the lung has limitations in evaluation of the health hazard of materials that are inhaled in the environment. The instillation of collected non-volatile material could not accurately mimic the particle size–dependent deposition pattern of inhaled PM. The comparative utility of dosing by inhalation and instillation has been reviewed (Driscoll et al. 2000), and although inhalation remains the “gold standard” for hazard assessment, instillation has proven useful for comparing effects among samples and screening for potential cause–effect relationships. Exposure of cultured cells is another alternative to inhalation for comparative toxicity screening, but work preceding the present analysis demonstrated that lung responses to instilled samples and responses of cultured epithelial cells and lung macrophages to the same samples gave quite different sample rankings (Seagrave et al. 2003). Compared with cell culture, lung instillation was considered the more relevant approach for identifying potential public health hazards. In view of the difficulty, cost, and time requirements of conducting inhalation exposures to a wide range of vehicle emissions, the study provided a test of the utility of a practical, albeit limited, approach to identifying chemical composition–toxicity associations warranting closer examination.

However good the models developed from the present sample set might be, caution must be exercised in extrapolating these results broadly to all gasoline and diesel engine emissions. The concordance of the present results for mutagenicity with pre-existing information on the importance of nitrogenated PAHs in different combustion emissions (e.g., Lewtas et al. 1992) suggests that the mutagenicity model might be broadly applicable to normal- and high-emitting gasoline and diesel engines and lends confidence that the lung toxicity results are also likely to be valid beyond this sample set. However, it is clear that lung toxicity was driven largely by the coincident differences in composition and toxicity between the samples from high-emitting and normal-emitting vehicles. The finding that lubricating oil tracers were highly associated with lung toxicity in this sample set does not necessarily mean that oil emissions would be the major determinant of lung toxicity in all engine emissions, and especially among emissions from engines having low oil consumption. The addition of more samples to the analysis, and especially samples differing even more markedly in composition, would bolster confidence in the results and their applicability across a broader spectrum of engine emissions. Regardless, the present results strongly indicate that attention should be given to oil-derived as well as fuel-derived emissions and suggest that as total emissions from fuel combustion continue to fall, oil-derived emissions could contribute relatively more to any residual health hazards.

There is little information on the effects of motor oil in the lung. Subchronic inhalation exposure of rats to high concentrations of aerosolized petroleum oils, including a formulation representing unused motor oil, produced only modest toxicity (Dalbey 2001). It is likely that the toxicity of motor oil increases with use. Zielinska et al. (2004) analyzed the composition of fuel and crankcase oil from the vehicles used in the present study. They reported that diesel fuel was enriched in light and semivolatile PAHs compared with gasoline fuel. In contrast, used oil from the gasoline-powered vehicles in this study was enriched in PAHs, including heavy, particle-phase PAHs, compared with used diesel oil. Lubricating oil in the gasoline vehicles apparently serves as a “sink” for the partitioning of combustion- or fuel-derived components; thus, it is important to consider the time in use of oil in studies of the contribution of oil components to the toxicity of engine emissions. Only one study has investigated the toxicity of used motor oil; Costa and Amdur (1979) reported a 28% increase in pulmonary resistance in guinea pigs exposed to used motor oil, but the variability in the pulmonary measurements rendered the difference from control animals insignificant. Clearly, more work needs to be done to investigate the toxicity of used motor oil as it is emitted in motor vehicle emissions.

A final caveat is that the statistical composition–response associations resulting from this work do not prove causality. There is considerable information indicating that nitro-PAHs cause mutations in bacteria, but there is little information on the effects of hopanes and steranes in the lung. It is possible that these putative agents could have covaried in mass concentration with unknown proximal causal species, rather than actually causing the responses. Although the composition of the samples was determined in detail, the measured mass by organic speciation accounted for only a small percentage (average ~10%) of the total SVOC + PM mass. Additional samples having different toxicity and chemical composition would strengthen the confidence in the observed associations. The causality of specific chemical classes or components of exhaust can be examined in complementary studies, including exposure to inhaled emissions containing different contributions from crankcase oil, “doping” samples with the putative causal agents, and/or progressive fractionation and testing of samples (i.e., bioassay directed fractionation).

The bioassay-directed fractionation approach may be useful for confirming and further evaluating the components that correlate with pulmonary toxicity. However, an important consideration in applying bioassay-directed fractionation for pulmonary toxicity is the much larger effort and cost of the *in vivo* assays relative to the simpler, less expensive bacterial mutagenicity assays that have been used. As mentioned above, *in vitro* testing with lung cells ranked the samples quite differently from the *in vivo* results (Seagrave et al. 2003). Because *in vivo* toxicity should be more relevant to human health hazard than *in vitro* results, it appears unlikely that biodirected fractionation for nonmutagenic lung toxicity can be done using *in vitro* assays.

## Conclusions

Despite its several limitations, this study provides important insights into the physical-chemical components of engine emissions that most strongly influence the toxicity of inhaled emissions. We extend the previous conclusion (Seagrave et al. 2002) that high-emitting vehicles contribute disproportionately to the health hazards of engine emissions, to conclude now that crankcase oil–derived, particle-associated organic compounds may contribute strongly to the inflammatory effects of inhaled emissions from high-emitting vehicles. Importantly, the chemicals most closely associated with pulmonary toxicity were different from the chemicals (e.g., nitro-PAHs and oxy-PAHs such as quinones) that were associated with bacterial mutagenicity. This is especially important considering the small amount of information available on chemicals that are associated with pulmonary toxicity. Further work is warranted to confirm the causality of specific classes and compounds, to confirm that oil-derived components are important to the toxicity of inhaled (as well as instilled) emissions, and to determine the relative importance of oil- versus fuel-derived components to the health hazards of emissions from a broader range of normal- and high-emitting vehicles. Moreover, we conclude that the PCA/PLS analytical strategy shows promise for disentangling composition–response associations, even when the exposures are extremely complex, the number of exposures is limited, and multiple responses are measured. In such situations, the success of the approach hinges on the extent to which composition and response variables can be lumped into covarying groups such that predictive models require a number of principal components substantially less than the number of exposures.

## Figures and Tables

**Table 1:** Summary description of engine samples, chemical measurements, and toxicity measurements.

Exhaust samples*^a^*
Gasoline
G
G_30_
WG
BG
Diesel
Diesel
D_30_
HD
Chemical measurements (by class)*^b^*
Total particle and SVOC mass
Inorganic ions
Carbon (organic, elemental, thermal fractions)
Transition metals
Other metals
Metalloids
Nonmetal elements
Bicyclic or two-ring PAH organic compounds
Tricyclic or three-ring PAH organic compounds
Tetracyclic or four-ring PAH organic compounds
Five-ring PAH organic compounds
Six-ring PAH organic compounds
Seven-ring PAH organic compounds
Oxygenated PAH organic compounds (including quinones)
Sulfur-containing PAHs
Nitro-PAH organic compounds
Hopane/sterane organic compounds (unique to oil)
Toxicity measurements*^c^*
Lung toxicity
Inflammation potency estimates
Lavage macrophages
Lavage neutrophils
Total lavage leukocytes
Histopathologic inflammation
Macrophage inflammatory protein-2
Cytotoxicity potency estimates
Lactate dehydrogenase
Lavage protein
Histopathologic tissue injury
Parenchymal change potency estimates
Histopathologic structural remodeling
General toxicity potency estimates
Total histopathology
Lung weight as percentage of body weight
Bacterial mutagenicity
Mutagenicity potency estimates
TA98 − S9
TA98 + S9
TA100 − S9
TA100 + S9

a Described by Whitney (2000).

b Described by Zielinska et al. (2004).

c Described by Seagrave et al. (2002).

**Table 2:** Chemical/physical variables (X-matrix) included in the final PLS model for lung toxicity.

Chemical/physical component	Name used for loading plot
Particulate mass	PM
Semivolatile organic mass	SVOC
Nitrate	NO_3_
Sulfate	SO_4_
Ammonium	NH_4_
Particle organic carbon mass	Particle organic carbon
Elemental carbon	EC
Total carbon	TC
1st carbon thermal fraction	O1TC
2nd carbon thermal fraction	O2TC
3rd carbon thermal fraction	O3TC
4th carbon thermal fraction	O4TC
5th carbon thermal fraction	OPTC
6th carbon thermal fraction	E1TC
7th carbon thermal fraction	E2TC
8th carbon thermal fraction	E3TC
Transition metals	Trans. met
Alkali earth metals	Earth met
Metalloids	Metalloids
Nonmetal elements	Non-met
Group 3A metals	3A metals
Group 4A metals	4A metals
Total PAHs	Total PAH
SVOC PAHs	SVOC PAH
PM PAHs	PM PAH
Two-ring PAHs	2-ring PAH
Three-ring PAHs	3-ring PAH
Four-ring PAHs	4-ring PAH
Five-ring PAHs	5-ring PAH
> Five-ring PAHs	> 5-ring PAH
Nitro-PAHs	Nitro-PAH
Oxygenated PAHs	Oxy-PAH
C27-20S-13β(H),17α(H)-diasterane	S1
C27-20R-13β(H),17α(H)-diasterane	S2
C27-20S-13α(H),17β(H)-diasterane	S3
C27-20R-13α(H),17β(H)-diasterane	S4
C28-20S-13β(H),17α(H)-diasterane	S5
C27-20S-5α(H),14α(H)-cholestane	S6
C27-20R-5α(H),14β(H)-cholestane	S7
C27-20S-5α(H),14β(H),17β(H)-cholestane	S8
C27-20R-5α(H),14α(H),17α(H)-cholestane	S9
C28-20S-5α(H),14α(H),17α(H)-ergostane	S10
C28-20R-5α(H),14β(H),17β(H)-ergostane	S11
C29-20R-13α(H),17β(H)-diasterane	S12
C27-tetracyclic terpane	S13
C28-20R-5α(H),14α(H),17α(H)-ergostane	S14
C27-tetracyclic terpane II	S15
C28-tetracyclic terpane	S16
C29-20S-5α(H),14α(H),17α(H)-stigmastane	S17
C28-tetracyclic terpane II	S18
C29-20R-5α(H),14β(H),17β(H)-stigmastane	S19
C29-20S-5α(H),14β(H),17β(H)-stigmastane	S20
18α(H),21β(H)-22,29,30-trisnorhopane	H1
17α(H),18α(H),21β(H)-25,28,30-trisnorhopane	H2
C29-20R-5α(H),14α(H),17α(H)-stigmastane	H3
17α(H),21β(H)-22,29,30-trisnorhopane	H4
17α(H),18α(H),21β(H)-28,30-bisnorhopane	H5
17α(H),21β(H)-30-norhopane	H6
18α(H),21β(H)-30-norneohopane	H7
17α(H),21β(H)-hopane	H8
17β(H),21α(H)-hopane	H9
22S-17α(H),21β(H)-30-homohopane	H10
22R-17α(H),21β(H)-30-homohopane	H11
17β(H),21β(H)-hopane	H12
22S-17α(H),21β(H)-30,31-bishomohopane	H13
22R-17α(H),21β(H)-30,31-bishomohopane	H14
22S-17α(H),21β(H)-30,31,32-trishomohopane	H15
22R-17α(H),21β(H)-30,31,32-trishomohopane	H16

**Table 3:** Chemical/physical variables (X-matrix) used for final PLS model of mutagenicity.

Oxygenated PAH organic compounds
9-Fluorenone
Xanthone
Acenaphthenequinone
Perinaphthenone
Anthraquinone
9-Anthraldehyde
Benz[*a*]anthracene-7,12-dione
Nitro-PAH organic compounds
1-Nitronaphthalene
2-Nitronaphthalene
2-Methyl-1-nitronaphthalene
α-Methyl-1-nitronaphthalene
β-Methyl-1-nitronaphthalene
2-Nitrobiphenyl
4-Nitrobiphenyl
5-Nitroacenaphthene
9-Nitroanthracene
2-Nitrofluoranthene
3-Nitrofluoranthene
1-Nitropyrene
7-Nitrobenz[*a*]anthracene
6-Nitrochrysene
6-Nitrobenzo[*a*]pyrene

**Figure A2-1:**
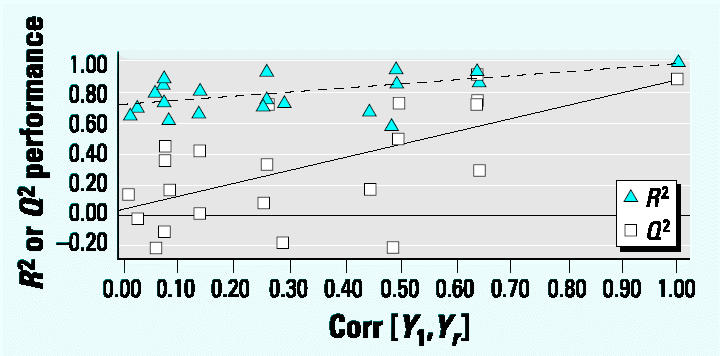
Scatter plot of corr(*Y*,*Y_r_*) versus *Q*^2^[*Y_r_*]. The near zero *y*-intercept of the line plotted through the *Q*^2^ values confirms that the base model (right) is not due to random statistical associations, by showing that changes in the structure of the data decrease the performance of the model. High *R*^2^ for nearly all of the model permutations illustrates the need for the *Q*^2^ diagnostic because correlation (*R*^2^) occurs under several conditions without good model performance.

## Appendix

**Appendix 1:** Entire compositional data set presented as weight fraction.

	BG	WG	HD	G	G_30_	D	D_30_
Total PM and SVOC							
Particle mass	0.288877	0.689480	0.628759	0.162128	0.295583	0.717567	0.693145
SVOC mass	0.711123	0.310520	0.371241	0.837872	0.704417	0.282433	0.306855
Inorganic ions							
Chloride	0.000891	0.000661	0.000017	0.001062	0.001106	0.000637	0.000000
Nitrate	0.000294	0.001022	0.000345	0.000273	0.000422	0.000254	0.000585
Sulfate	0.002643	0.002143	0.003073	0.004721	0.013193	0.174678	0.049407
Ammonium	0.001337	0.001251	0.001060	0.004087	0.009031	0.003130	0.001317
Sodium	0.000099	0.000227	0.000516	0.000001	0.000000	0.000185	0.000117
Carbon							
Particle organic carbon mass	0.195937	0.657895	0.488888	0.084029	0.100675	0.141816	0.461659
Elemental carbon	0.044261	0.020826	0.130267	0.040269	0.148137	0.348723	0.123590
Total carbon	0.207543	0.569073	0.537674	0.110293	0.232034	0.466659	0.508301
Carbon splits							
O1TC	0.087617	0.197504	0.267370	0.031775	0.045685	0.056771	0.099821
O2TC	0.066411	0.297777	0.140208	0.014997	0.018800	0.025543	0.031097
O3TC	0.012191	0.032741	0.033965	0.013365	0.014883	0.029318	0.038484
O4TC	0.009070	0.009965	0.009971	0.010214	0.013101	0.026178	0.031534
OPTC	0.020649	0.119907	0.037376	0.013678	0.008207	0.004008	0.260722
E1TC	0.059643	0.110620	0.035683	0.043630	0.053664	0.196258	0.217269
E2TC	0.001662	0.009845	0.125607	0.007894	0.101237	0.155739	0.123341
E3TC	0.000164	0.000285	0.000121	0.000233	0.000108	0.000068	0.000244
Transition metals							
Titanium	0.000008	0.000000	0.000000	0.000016	0.000004	0.000027	0.000033
Vanadium	0.000000	0.000004	0.000002	0.000003	0.000018	0.000012	0.000000
Chromium	0.000088	0.000009	0.000020	0.000051	0.000107	0.000585	0.000473
Manganese	0.000161	0.000008	0.000003	0.000064	0.000086	0.000078	0.000108
Iron mass	0.022771	0.000475	0.000280	0.012046	0.012934	0.011622	0.026206
Cobalt	0.000002	0.000003	0.000006	0.000000	0.000000	0.000005	0.000022
Nickel	0.000054	0.000002	0.000005	0.000029	0.000056	0.000262	0.000311
Copper	0.000352	0.000049	0.000002	0.000119	0.000110	0.000076	0.000113
Zinc	0.002677	0.000536	0.000389	0.001604	0.001206	0.000879	0.001239
Yttrium	0.000006	0.000007	0.000000	0.000002	0.000003	0.000006	0.000011
Zirconium	0.000030	0.000018	0.000000	0.000024	0.000054	0.000010	0.000021
Molybdenum	0.000017	0.000000	0.000000	0.000002	0.000004	0.000000	0.000000
Palladium	0.000022	0.000000	0.000003	0.000001	0.000001	0.000000	0.000007
Silver	0.000021	0.000000	0.000035	0.000009	0.000000	0.000065	0.000000
Cadmium	0.000012	0.000000	0.000000	0.000001	0.000010	0.000001	0.000000
Lanthanum	0.000000	0.000000	0.000000	0.000020	0.000000	0.000022	0.000000
Gold	0.000020	0.000004	0.000025	0.000016	0.000018	0.000020	0.000039
Mercury	0.000002	0.000009	0.000021	0.000002	0.000001	0.000002	0.000000
Other metals							
Magnesium	0.000831	0.000449	0.000110	0.000296	0.000133	0.000146	0.000140
Aluminum mass	0.000254	0.000000	0.000000	0.000292	0.000259	0.000522	0.001545
Potassium	0.000069	0.000000	0.000000	0.000146	0.000087	0.000057	0.000027
Calcium	0.001574	0.000304	0.000668	0.001825	0.001369	0.001708	0.002423
Gallium	0.000003	0.000006	0.000018	0.000000	0.000001	0.000001	0.000006
Rubidium	0.000004	0.000008	0.000005	0.000002	0.000003	0.000005	0.000004
Strontium	0.000000	0.000003	0.000008	0.000004	0.000002	0.000005	0.000008
Indium	0.000028	0.000101	0.000107	0.000012	0.000005	0.000027	0.000023
Barium	0.000064	0.000234	0.000000	0.000101	0.000033	0.000249	0.000000
Thallium	0.000000	0.000025	0.000012	0.000005	0.000003	0.000017	0.000013
Lead	0.004843	0.000034	0.000046	0.000264	0.000397	0.000041	0.000036
Uranium	0.000001	0.000007	0.000021	0.000004	0.000000	0.000005	0.000000
Metalloids							
Silicon mass	0.005000	0.000951	0.000918	0.007752	0.002787	0.003295	0.004616
Arsenic	0.000000	0.000000	0.000000	0.000001	0.000002	0.000004	0.000000
Tin	0.000050	0.000064	0.000033	0.000032	0.000023	0.000024	0.000047
Antimony	0.000006	0.000031	0.000172	0.000009	0.000004	0.000000	0.000036
Nonmetal elements							
Sulfur	0.002354	0.001825	0.001508	0.001716	0.002100	0.028206	0.018411
Chlorine	0.000726	0.000008	0.000016	0.000236	0.000601	0.000000	0.000000
Phosphorous	0.001334	0.000274	0.000152	0.000978	0.000591	0.000139	0.000545
Selenium	0.000001	0.000002	0.000006	0.000003	0.000002	0.000010	0.000008
Bromine	0.000028	0.000004	0.000003	0.000004	0.000001	0.000010	0.000000
Bicyclic or two-ring PAH Organic Compounds							
Naphthalene	0.003309	0.003459	0.000626	0.045334	0.003114	0.003792	0.011416
2-Menaphthalene	0.001595	0.002445	0.000997	0.017146	0.002901	0.002079	0.003719
1-Menaphthalene	0.001034	0.002018	0.001099	0.016027	0.002357	0.002054	0.003757
2,6 + 2,7-Dimenaphthalene	0.003518	0.002303	0.000676	0.008528	0.006724	0.001017	0.001218
1,6 + 1,3+1,7-Dimethylnaphthalene	0.004080	0.003833	0.001158	0.012884	0.009602	0.001726	0.002036
2,3 + 1,4+1,5-Dimenaphthalene	0.000883	0.001516	0.000371	0.004195	0.003289	0.000562	0.000709
1,2-Dimethylnaphthalene	0.000401	0.000984	0.000195	0.001914	0.001984	0.000248	0.000252
1-Ethyl-2-methylnaphthalene	0.002846	0.002836	0.000218	0.004541	0.004886	0.000375	0.000480
Biphenyl	0.001059	0.000927	0.000379	0.005099	0.002949	0.000635	0.000976
2-Methylbiphenyl	0.000169	0.000157	0.000102	0.000821	0.000306	0.000167	0.000178
3-Methylbiphenyl	0.000233	0.001011	0.000539	0.005404	0.001538	0.000921	0.001338
4-Methylbiphenyl	0.000104	0.000519	0.000207	0.002470	0.000749	0.000355	0.000613
Bibenzyl	0.000792	0.004328	0.000068	0.006136	0.004549	0.000701	0.001061
α-Trimethylnaphthalene	0.000427	0.001258	0.000304	0.004647	0.002628	0.000666	0.001060
1-Ethyl-2-methylnaphthalene	0.014773	0.003354	0.000028	0.039013	0.033539	0.000881	0.001511
β-Trimethylnaphthalene	0.000285	0.000986	0.000481	0.004565	0.002411	0.000699	0.001057
γ-Trimethylnaphthalene	0.000177	0.000925	0.000336	0.004009	0.001965	0.000616	0.000953
2-Ethyl-1-methylnaphthalene	0.000020	0.000114	0.000009	0.000169	0.000150	0.000014	0.000024
ɛ-Trimethylnaphthalene	0.000111	0.000475	0.000296	0.002788	0.001094	0.000414	0.000658
f-Trimethylnaphthalene	0.000119	0.000455	0.000258	0.002878	0.001180	0.000425	0.000666
2,3,5-Trimethylnaphthalene	0.000172	0.001039	0.000509	0.005509	0.002367	0.000790	0.001268
2,4,5-Trimethylnaphthalene	0.000057	0.000399	0.000054	0.001224	0.000795	0.000092	0.000252
j-Trimethylnaphthalene	0.000048	0.000306	0.000125	0.001293	0.000505	0.000202	0.000303
1,4,5-Trimethylnaphthalene	0.000644	0.000408	0.000065	0.002682	0.002074	0.000288	0.000222
1,2,8-Trimethylnaphthalene	0.000295	0.000213	0.000011	0.001438	0.001124	0.000127	0.000132
Tricyclic or three-ring PAH organic compounds							
Acenaphthylene	0.002691	0.004103	0.000115	0.008941	0.025590	0.000486	0.002132
Acenaphthene	0.000280	0.000414	0.000046	0.001136	0.001579	0.000107	0.000242
Fluorene	0.000879	0.003382	0.000238	0.012595	0.008075	0.000760	0.003204
Phenanthrene	0.001121	0.001538	0.000546	0.008017	0.008055	0.000650	0.001958
α-Methylfluorene	0.000187	0.000616	0.000218	0.002117	0.000878	0.000291	0.000519
1-Methylfluorene	0.000126	0.000471	0.000166	0.002054	0.000583	0.000312	0.000509
β-Methylfluorene	0.000062	0.000177	0.000037	0.000477	0.000206	0.000082	0.000117
γ-Methylphenanthrene	0.000159	0.000513	0.000238	0.002131	0.000709	0.000260	0.000519
2-Methylphenanthrene	0.000188	0.000629	0.000238	0.002574	0.000856	0.000351	0.000633
γ-Methylphenanthrene	0.000176	0.000601	0.000135	0.001923	0.000862	0.000196	0.000473
1-Methylphenanthrene	0.000147	0.000491	0.000110	0.001590	0.000521	0.000242	0.000383
3,6-Dimethylphenanthrene	0.000031	0.000135	0.000058	0.000637	0.000145	0.000085	0.000160
α-Dimethylphenanthrene	0.000052	0.000234	0.000071	0.000979	0.000184	0.000123	0.000246
β-Dimethylphenanthrene	0.000029	0.000138	0.000035	0.000632	0.000108	0.000087	0.000162
γ-Dimethylphenanthrene	0.000116	0.000606	0.000125	0.001837	0.000448	0.000227	0.000460
1,7-Dimethylphenanthrene	0.000073	0.000392	0.000057	0.000940	0.000268	0.000103	0.000231
d-Dimethylphenanthrene	0.000024	0.000131	0.000026	0.000567	0.000103	0.000070	0.000145
ɛ-Dimethylphenanthrene	0.000053	0.000353	0.000037	0.000803	0.000248	0.000091	0.000188
Anthracene	0.000228	0.000438	0.000041	0.001107	0.001244	0.000046	0.000258
9-Methylanthracene	0.000028	0.000098	0.000001	0.000129	0.000052	0.000021	0.000029
Retene	0.000010	0.000005	0.000003	0.000032	0.000010	0.000006	0.000008
Tetracyclic or four-ring PAH organic compounds							
2,3-Benzofluorene	0.000156	0.001283	0.000003	0.000957	0.001241	0.000013	0.000192
Fluoranthene	0.000454	0.000778	0.000026	0.002844	0.003514	0.000097	0.000688
Pyrene	0.000344	0.000409	0.000058	0.002116	0.001901	0.000045	0.000548
1-Methylfluorene + a-methylfluorene	0.000002	0.000012	0.000001	0.000008	0.000007	0.000001	0.000000
b-Methylpyrene + b-methylfluorene	0.000122	0.000851	0.000009	0.000769	0.000654	0.000031	0.000164
c-Methylpyrene + c-methylfluorene	0.000084	0.000620	0.000002	0.000462	0.000573	0.000009	0.000094
4-Methylpyrene	0.000027	0.000109	0.000010	0.000227	0.000084	0.000006	0.000058
1-Methylpyrene	0.000023	0.000127	0.000006	0.000196	0.000078	0.000003	0.000050
Benzo[*c*]phenanthrene	0.000032	0.000101	0.000001	0.000207	0.000216	0.000006	0.000052
Benz[*a*]anthracene	0.000065	0.000173	0.000002	0.000275	0.000293	0.000004	0.000067
7-Methylbenz[*a*]anthracene	0.000001	0.000002	0.000000	0.000002	0.000003	0.000000	0.000000
Chrysene	0.000093	0.000250	0.000006	0.000849	0.000516	0.000022	0.000227
5+6-Methylchrysene	0.000008	0.000055	0.000001	0.000059	0.000031	0.000027	0.000015
Five-ring PAH organic compounds							
Benzo[*b*+*j*+*k*]FL	0.000071	0.000137	0.000003	0.000449	0.000326	0.000008	0.000122
7-Methylbenzo[*a*]pyrene	0.000001	0.000003	0.000000	0.000007	0.000006	0.000003	0.000002
Benzo[*e*]pyrene	0.000031	0.000090	0.000002	0.000225	0.000147	0.000003	0.000060
Perylene	0.000010	0.000023	0.000000	0.000010	0.000062	0.000001	0.000002
Benzo[*a*]pyrene	0.000038	0.000099	0.000001	0.000089	0.000225	0.000028	0.000022
Six-ring PAH organic compounds							
Indeno[*123-cd*]pyrene	0.000035	0.000071	0.000001	0.000204	0.000204	0.000002	0.000056
Benzo[*ghi*]perylene	0.000104	0.000107	0.000002	0.000276	0.000279	0.000003	0.000074
Dibenz[*ah*+*ac*]anthracene	0.000004	0.000015	0.000000	0.000032	0.000024	0.000000	0.000009
Seven-ring PAH organic compounds							
Coronene	0.000056	0.000007	0.000001	0.000078	0.000103	0.000002	0.000020
Oxygenated PAH organic compounds							
9-Fluorenone	0.007703	0.001677	0.000001	0.012351	0.020705	0.000564	0.000399
Xanthone	0.000000	0.000060	0.000071	0.000008	0.000099	0.000048	0.000002
Acenaphthenequinone	0.000000	0.000018	0.000002	0.000000	0.000000	0.000016	0.000000
Perinaphthenone	0.000000	0.000000	0.000020	0.000000	0.000000	0.000000	0.000000
Anthraquinone	0.000103	0.000466	0.000001	0.000712	0.000507	0.000034	0.000140
9-Anthraldehyde	0.000033	0.000000	0.000035	0.000309	0.000056	0.000112	0.000085
Benz[*a*]anthracene-7,12-dione	0.000003	0.000050	0.000000	0.000035	0.000055	0.000004	0.000009
Sulfur-containing PAH							
Benzonaphthothiopene	0.000005	0.000064	0.000001	0.000091	0.000012	0.000005	0.000024
Nitro PAH organic compounds							
1-Nitronaphthalene	0.000012	0.000007	0.000012	0.000029	0.000008	0.000012	0.000013
2-Nitronaphthalene	0.000005	0.000002	0.000002	0.000016	0.000011	0.000004	0.000005
2-Methyl-1-nitronaphthalene	0.000001	0.000001	0.000000	0.000000	0.000001	0.000008	0.000001
α-Methyl-1-nitronaphthalene	0.000001	0.000001	0.000000	0.000001	0.000001	0.000001	0.000000
β-Methyl-1-nitronaphthalene	0.000003	0.000008	0.000003	0.000003	0.000003	0.000005	0.000003
2-Nitrobiphenyl	0.000000	0.000000	0.000000	0.000000	0.000000	0.000000	0.000000
4-Nitrobiphenyl	0.000005	0.000002	0.000012	0.000003	0.000002	0.000003	0.000007
5-Nitroacenaphthene	0.000000	0.000000	0.000000	0.000000	0.000000	0.000000	0.000000
9-Nitroanthracene	0.000000	0.000000	0.000000	0.000001	0.000002	0.000002	0.000002
2-Nitrofluoranthene	0.000000	0.000000	0.000000	0.000000	0.000000	0.000000	0.000000
3-Nitrofluoranthene	0.000000	0.000000	0.000000	0.000000	0.000000	0.000000	0.000000
1-Nitropyrene	0.000001	0.000001	0.000002	0.000001	0.000000	0.000013	0.000010
7-Nitrobenz[*a*]anthracene	0.000000	0.000001	0.000000	0.000000	0.000000	0.000000	0.000001
6-Nitrochrysene	0.000002	0.000000	0.000001	0.000006	0.000001	0.000002	0.000003
6-Nitrobenzo[*a*]pyrene	0.000000	0.000000	0.000001	0.000000	0.000000	0.000001	0.000005
Hopane/sterane organic compounds							
C27-20S-13β(H),17α(H)-diasterane	0.000010	0.000011	0.000010	0.000002	0.000002	0.000007	0.000004
C27-20R-13β(H),17α(H)-diasterane	0.000007	0.000006	0.000005	0.000002	0.000001	0.000006	0.000002
C27-20S-13α(H),17β(H)-diasterane	0.000003	0.000003	0.000003	0.000001	0.000000	0.000002	0.000001
C27-20R-13α(H),17β(H)-diasterane	0.000004	0.000006	0.000004	0.000001	0.000001	0.000003	0.000001
C28-20S-13β(H),17α(H)-diasterane	0.000005	0.000004	0.000001	0.000001	0.000001	0.000002	0.000001
C27-20S-5α(H),14α(H)-cholestane	0.000019	0.000007	0.000001	0.000001	0.000002	0.000006	0.000003
C27-20R-5α(H),14β(H)-cholestane	0.000019	0.000014	0.000018	0.000002	0.000002	0.000010	0.000005
C27-20S-5α(H),14β(H),17β(H)-cholestane	0.000007	0.000009	0.000012	0.000001	0.000002	0.000005	0.000003
C27-20R-5α(H),14α(H),17α(H)-cholestane	0.000019	0.000022	0.000020	0.000003	0.000004	0.000007	0.000008
C28-20S-5α(H),14α(H),17α(H)-ergostane	0.000003	0.000005	0.000003	0.000001	0.000001	0.000003	0.000002
C28-20R-5α(H),14β(H),17β(H)-ergostane	0.000007	0.000008	0.000010	0.000001	0.000001	0.000007	0.000003
C29-20R-13α(H),17β(H)-diasterane	0.000008	0.000016	0.000023	0.000002	0.000003	0.000006	0.000005
C27-Tetracyclic terpane	0.000010	0.000016	0.000037	0.000005	0.000004	0.000014	0.000007
C28-20R-5α(H),14α(H),17α(H)-ergostane	0.000004	0.000007	0.000008	0.000001	0.000001	0.000004	0.000002
C27-Tetracyclic terpane	0.000003	0.000002	0.000000	0.000001	0.000001	0.000004	0.000003
C28-Tetracyclic terpane	0.000009	0.000007	0.000144	0.000004	0.000004	0.000009	0.000005
C29-20S-5α(H),14α(H),17α(H)-stigmastane	0.000005	0.000012	0.000014	0.000001	0.000002	0.000007	0.000004
C28-Tetracyclic terpane	0.000003	0.000019	0.000001	0.000001	0.000001	0.000005	0.000003
C29-20R-5α(H),14β(H),17β(H)-stigmastane	0.000010	0.000015	0.000021	0.000001	0.000003	0.000008	0.000006
C29-20S-5α(H),14β(H),17β(H)-stigmastane	0.000006	0.000010	0.000013	0.000001	0.000002	0.000006	0.000003
18α(H),21β(H)-22,29,30-trisnorhopane	0.000002	0.000002	0.000009	0.000000	0.000000	0.000001	0.000001
17α(H),18α(H),21β(H)-25,28,30-trisnorhopane	0.000001	0.000002	0.000006	0.000000	0.000000	0.000001	0.000001
C29-20R-5α(H),14α(H),17α(H)-stigmastane	0.000008	0.000015	0.000016	0.000001	0.000003	0.000007	0.000004
17α(H),21β(H)-22,29,30-trisnorhopane	0.000042	0.000083	0.000106	0.000012	0.000016	0.000056	0.000030
17α(H),18α(H),21β(H)-28,30-bisnorhopane	0.0000010	0.0000022	0.0000019	0.0000001	0.0000002	0.0000008	0.0000004
17α(H),21β(H)-30-norhopane	0.0000319	0.0000690	0.0000000	0.0000073	0.0000095	0.0000412	0.0000196
18α(H),21β(H)-30-norneohopane	0.0000040	0.0000099	0.0000027	0.0000006	0.0000003	0.0000030	0.0000031
17α(H),21β(H)-hopane	0.0000029	0.0000066	0.0000076	0.0000003	0.0000009	0.0000030	0.0000014
17β(H),21α(H)-hopane	0.0000020	0.0000023	0.0000081	0.0000002	0.0000007	0.0000023	0.0000014
22S-17α(H),21β(H)-30-homohopane	0.0000146	0.0000345	0.0000514	0.0000027	0.0000046	0.0000187	0.0000088
22R-17α(H),21β(H)-30-homohopane	0.0000090	0.0000258	0.0000351	0.0000018	0.0000035	0.0000129	0.0000067
17β(H),21β(H)-hopane	0.0000031	0.0000123	0.0000040	0.0000004	0.0000010	0.0000043	0.0000019
22S-17α(H),21β(H)-30,31-bishomohopane	0.0000072	0.0000199	0.0000544	0.0000011	0.0000025	0.0000094	0.0000045
22R-17α(H),21β(H)-30,31-bishomohopane	0.0000045	0.0000136	0.0000342	0.0000009	0.0000014	0.0000070	0.0000035
22S-17α(H),21β(H)-30,31,32-trishomohopane	0.0000044	0.0000155	0.0000156	0.0000006	0.0000015	0.0000055	0.0000023
22R-17α(H),21β(H)-30,31,32-trishomohopane	0.0000032	0.0000098	0.0000093	0.0000004	0.0000010	0.0000036	0.0000016

## Appendix 2. Evaluation and Validation of PLS Model

### Methods for evaluation of statistical modeling results.

The PLS model results were evaluated based on indices of goodness of fit (*R*^2^) and prediction capacity (*Q*^2^). The *R*^2^ goodness of fit coefficient is analogous to the multiple regression correlation coefficient (squared Pearson product-moment correlation between observed and predicted Y-values). Although this index provides insight into the strength of the observed association between model-predicted and observed health outcomes, it is not necessarily a reliable measure of the predictive capacity of models. *Q*^2^ is calculated for this purpose. Based on the cross-validation technique described by Wold (1978), *Q*^2^ assesses the model’s ability to predict health outcomes for each individual sample when that sample is not used in the PLS model. The *Q*^2^ goodness of prediction parameter is similar to *R*^2^ in that it is based on the sums of squares of prediction errors. However, unlike the prediction errors used in calculating *R*^2^, the prediction errors for *Q*^2^ are independent of the prediction itself, rendering *Q*^2^ a more reliable index of prediction performance.

Both *R*^2^ and *Q*^2^ values were calculated for model predictions on the original data matrix as well as for 20 random orderings of the Y (health outcomes) data matrix, while keeping the X-matrix (emissions sample composition) fixed. As the randomly reordered Y-matrix (*Y**_r_*) changes, the correlation between the original Y-values and reordered Y-values, (i.e., corr[*Y*_1_,*Y**_r_*]), decreases to smaller and smaller values. If there is an underlying systematic (nonrandom) relationship between the Y- and X-matrices, a PLS model constructed on the randomly reordered Y-values (i.e., *Y**_r_*) would be expected to exhibit predictive power that decreases (decreased *Q*^2^) as corr[*Y*_1_,*Y**_r_*] decreases. If this does not occur, and the predictive capability of a model based on random pairings of the health outcome data with their emission sample predictors is as good as the predictive capability of the model based on the observed pairing of health outcome and emissions sample data, there is good evidence that the model is based on chance as opposed to systematic (nonrandom) associations between the health outcome and emission composition. Thus, the test of model plausibility is based upon the examination of relationship between corr[*Y*_1_,*Y**_r_*] and the *Q*^2^ associated with reordered Y-values (i.e., *Q*^2^[*Y**_r_*]). If the model is capturing a systematic (nonrandom) relationship, the scatter plot of corr(*Y*_1_,*Y**_r_*) versus *Q*^2^[*Y**_r_*] should exhibit a linear relationship, and the estimated intercept should be near zero. The *R*^2^ value may be in the acceptable range even when the variation in the Y-matrix yields an unacceptable *Q*^2^, indicating that data exhibit correlations by chance but there are no clear differences in the associations between specific predictor variables and the dependent variables. This illustrates the importance of using the *Q*^2^ criterion with permutation testing of these models, which is not always conducted and reported in the literature.

The overriding difficulty in performing the present analysis was the large number of predictor (composition) variables and relatively smaller number of emission samples on which to examine health outcomes. Without some strategy to group (and reduce) the number of composition variables, the identification of systematic (nonrandom) relationships between the composition variables and health outcomes might prove impossible. The strategy for grouping predictor variables focused on composites (sums) of chemical classes or subclasses. This strategy allowed greater interpretive ability because the importance of specific classes of components could be identified. Compounds were also grouped because the lower number of predictor variables improved the model performance (*R*^2^, *Q*^2^). The problem with grouping compounds in a particular chemical class is that the assumption is made that the individual compounds within that group contribute equally to toxicity. If there are differences among the toxicity of individual components, combining them might mask the effects of the most important components. In addition, grouping compounds masks the differences in the concentrations of individual compounds among the group.

### Validation of statistical model, example.

Each iteration of the PLS model was systematically validated as described herein. This began with evaluation of the performance parameters (*R*^2^ and *Q*^2^, with 1.0 being perfect correlation or goodness of fit or prediction, respectively) of the base model, and was followed by validation by response permutation to ensure that the overall model was robust and not due to chance (random associations). As described, the final model plausibility was based on the relationship between corr[*Y*_1_,*Y**_r_*] and the *Q*^2^ associated with reordered response (in this study, response is toxicity) data. Here we give one example (Figure A2-1) of the result from the validation by response permutation for the PLS prediction of lavage protein. Twenty random variations (permutations) of the ordering of the compositional data were modeled by PLS, and the scatter plot of corr(*Y*_1_,*Y**_r_*) versus *Q*^2^[*Y**_r_*] (Figure A2-1) had an intercept near zero, indicating that the base model associations were non-random. An important point that is illustrated by this scatter plot is that each of the reordered Y-matrix models showed acceptable *R*^2^, even when there was poor *Q*^2^. This is important because models that rely on *R*^2^ alone may show correlations between variables that are actually random associations, and this would not be detected without validation using the *Q*^2^ criteria.

Each of the individual models and iterations of the groupings of the compositional variables was evaluated by this permutation of the Y-matrix. Only models where the slope of corr(*Y*_1_,*Y**_r_*) versus *Q*^2^[*Y**_r_*] was near zero were accepted and used to show associations between compositional components and lung or mutagenicity responses.

## References

[b1-ehp0112-001527] Arey J, Zielinska B, Atkinson R, Winer AM (1988). Formation of nitroarenes during ambient high-volume sampling. Environ Sci Technol.

[b2-ehp0112-001527] Chow JC, Watson JG, Crow D, Lowenthal DH, Merrifield T (2001). Comparison of IMPROVE and NIOSH carbon measurements. Aerosol Sci Technol.

[b3-ehp0112-001527] Costa DL, Amdur MO (1979). Respiratory response of guinea pigs to oil mists. Am Ind Hyg Assoc J.

[b4-ehp0112-001527] Dalbey WE (2001). Subchronic inhalation exposures to aerosols of three petroleum lubricants. Am Ind Hyg Assoc J.

[b5-ehp0112-001527] Driscoll KE, Costa DL, Hatch G, Henderson R, Oberdorster G, Salem H (2000). Intratracheal instillation as an exposure technique for the evaluation of respiratory tract toxicity: uses and limitations. Toxicol Sci.

[b6-ehp0112-001527] Eide I, Neverdal G, Thorvaldsen B, Grung B, Kvalheim OM (2002). Toxicological evaluation of complex mixtures by pattern recognition: correlating chemical fingerprints to mutagenicity. Environ Health Perspect.

[b7-ehp0112-001527] Eide I, Neverdal G, Thorvaldsen B, Shen H, Grung B, Kvalheim O (2001). Resolution of GC-MS data of complex PAC mixtures and regression modeling of mutagenicity by PLS. Environ Sci Technol.

[b8-ehp0112-001527] Jackson JE 1991. A User’s Guide to Principal Components. New York:John Wiley.

[b9-ehp0112-001527] Kettaneh-Wold N (1992). Analysis of mixture data with partial least squares. Chemom Intell Lab Syst.

[b10-ehp0112-001527] Kvalheim OM (1989). Model building in chemistry, a unified approach. Anal Chim Acta.

[b11-ehp0112-001527] Lewtas J, Lewis C, Zweidinger R, Stevens R, Cupitt L (1992). Sources of genotoxicity and cancer risk in ambient air. Pharmacogenetics.

[b12-ehp0112-001527] Maejima K, Tamura K, Nakajima T, Taniguchi Y, Saito S, Takenada H (2001). Effects of the inhalation of diesel exhaust, Kanto loam dust, or diesel exhaust without particles on immune responses in mice exposed to Japanese cedar (*Cryptomeria japonica*) pollen. Inhal Toxicol.

[b13-ehp0112-001527] Mauderly JL (2003). Health effects of air pollution: the struggle for context [Editorial]. Environ Progr.

[b14-ehp0112-001527] Maykut N, Kim E, Lewas J, Larson TV (2003). Source apportionment of PM_2.5_ at an urban IMPROVE site in Seattle, Washington. Environ Sci Technol.

[b15-ehp0112-001527] McDonald JD, Zielinska B, Sagebiel JC, McDaniel MR, Mousset-Jones P (2003). Source apportionment of airborne fine particulate matter in an underground mine. J Air Waste Manage Assoc.

[b16-ehp0112-001527] National Research Council 2001. National Research Council Particulate Matter Committee Report III: Research Priorities for Airborne Particulate Matter. Washington, DC:National Academies Press.

[b17-ehp0112-001527] Nel AE, Diaz-Sanchez D, Li N (2001). The role of particulate pollutants in pulmonary inflammation and asthma: evidence for the involvement of organic chemicals and oxidative stress. Curr Opin Pulm Med.

[b18-ehp0112-001527] Nicolai T, Carr D, Weiland SK, Duhme H, von Ehrenstein O, Wagner O (2003). Urban traffic and pollutant exposure related to respiratory outcomes and atopy in a large sample of children. Eur Respir J.

[b19-ehp0112-001527] Pearson RL, Wachtel J, Ebi KL (2000). Distance-weighted traffic density in proximity to a home is a risk factor for leukemia and other childhood cancers. J Air Waste Manage Assoc.

[b20-ehp0112-001527] Rogge WF, Hildemann LM, Mazurek MA, Cass GR (1993). Sources of fine organic aerosol. II. Noncatalyst and catalyst-equipped automobiles and heavy-duty diesel trucks. Environ Sci Technol.

[b21-ehp0112-001527] Samet JM, Zeger SL, Dominici F, Curriero F, Coursac I, Dockery DW (2000). The National Morbidity, Mortality, and Air Pollution Study. Part II: Morbidity and mortality from air pollution in the United States. Res Rep Health Eff Inst.

[b22-ehp0112-001527] Seagrave J, Mauderly JL, Seilkop SK (2003). *In vitro* relative toxicity screening of combined particulate and semivolatile organic fractions of gasoline and diesel engine emissions. J Toxicol Environ Health.

[b23-ehp0112-001527] Seagrave J, McDonald JD, Gigliotti AP, Nikula KJ, Seilkop SK, Gurevich M (2002). Mutagenicity and *in vivo* toxicity of combined particulate and semivolatile organic fractions of gasoline and diesel engine emissions. Toxicol Sci.

[b24-ehp0112-001527] Seagrave JC, Berger J, Zielinska B, Sagebiel J, McDonald JD, Mauderly JL (2001). Comparative acute toxicities of particulate matter and semivolatile organic compound fractions of traffic tunnel air. Toxicologist.

[b25-ehp0112-001527] Shah AB, Combes RD, Rowland IR (1990). Activation and detoxification of 1,8-dintropyrene by mammalian hepatic fractions in the *Salmonella* mutagenicity assay. Mutagenesis.

[b26-ehp0112-001527] Sjogren M, Li H, Banner C, Rafter J, Westerholm R, Rannug U (1996). Influence of physical and chemical characteristics of diesel fuels and exhaust emissions on biological effects of particle extracts: a multivariate statistical analysis of ten diesel fuels. Chem Res Toxicol.

[b27-ehp0112-001527] Van der Voet H (1994). Comparing the predictive accuracy of models using a simple randomization test. Chemom Intell Lab Syst.

[b28-ehp0112-001527] van Vliet P, Knape M, de Hartog J, Janssen N, Harssema H, Brunekreef B (1997). Motor vehicle exhaust and chronic respiratory symptoms in children living near freeways. Environ Res.

[b29-ehp0112-001527] Venn AJ, Lewis SA, Cooper M, Hubbard R, Britton J (2001). Living near a main road and the risk of wheezing illness in children. Am J Respir Crit Care Med.

[b30-ehp0112-001527] Watson JG, Zhu T, Chow JC, Engelbrecht J, Fujita EM, Wilson WE (2002). Receptor modeling application framework for particle source apportionment. Chemosphere.

[b31-ehp0112-001527] Wellenius GA, Coull BA, Godleski JJ, Koutrakis P, Okabe K, Savage ST (2003). Inhalation of concentrated ambient air particles exacerbates myocardial ischemia in conscious dogs. Environ Health Perspect.

[b32-ehp0112-001527] Whitney KA 2000. Collection of In-Use Mobile Source Emission Samples for Toxicity Testing. SwRI document 08.02602. Final report to the National Renewable Energy Laboratory. Golden, CO:National Renewable Energy Laboratory.

[b33-ehp0112-001527] Wold S (1978). Cross-validatory estimation of the number of components in factor and principal components models. Technometrics.

[b34-ehp0112-001527] Wold S, Kettaneh N, Thessem K (1996). Hierarchical multiblock PLS and PC models for easier model interpretation and as an alternative to variable selection. J Chemom.

[b35-ehp0112-001527] Wold S, Ruhe A, Wold H, Dunn WJ (1984). The collinearity problem in linear regression. The partial least squares (PLS) approach to generalized inverses. SIAM J Sci Stat Comput.

[b36-ehp0112-001527] Zielinska B, Sagebiel J, McDonald J, Whitney K, Lawson DR (2004). Emission rates and comparative composition of in-use diesel and gasoline fueled vehicle emissions. J Air Waste Manage Assoc.

